# Predictors of persistently elevated parathyroid hormone levels after parathyroidectomy: experience from a reference center

**DOI:** 10.1590/1806-9282.20250013

**Published:** 2025-10-17

**Authors:** Léo Canterle Dal Osto, Sofia Vacaro Macedo, Tayane Muniz Fighera

**Affiliations:** 1 Universidade Federal do Rio Grande do Sul, Program in Medical Sciences: Endocrinology – Porto Alegre (RS), Brazil.; 2 Hospital de Clínicas de Porto Alegre, Division of Internal Medicine – Porto Alegre (RS), Brazil.; 3 Universidade Federal do Rio Grande do Sul, Department of Internal Medicine – Porto Alegre (RS), Brazil.

**Keywords:** Parathyroid hormone, Hyperparathyroidism, Parathyroidectomy, Chronic kidney failure

## Abstract

**OBJECTIVE:**

Primary hyperparathyroidism is considered the third most common endocrine disorder. Moreover, persistently elevated levels of parathyroid hormone occur in up to 30% of patients undergoing parathyroidectomy. The aim of this study was to determine the prevalence of persistently elevated parathyroid hormone levels after parathyroidectomy and analyze its associated factors.

**METHODS:**

An anonymized medical record query was conducted, filtering patients who underwent parathyroidectomy at a tertiary hospital center in Southern Brazil from 2010 to 2019. Clinical, laboratory, and sociodemographic data were collected. The analyzed period ranged from 3 months prior to surgery and up to 18 months after surgery.

**RESULTS:**

A total of 261 patients with an average age of 56 (45–65) years and a body mass index of 25.53 (22.19–28.86) kg/m^2^ were included. Patients with normal parathyroid hormone levels after 18 months of surgery were compared to those with high parathyroid hormone levels in the same period. The latter had lower levels of median parathyroid hormone, minimum achieved parathyroid hormone, and higher values of vitamin D. The two groups showed no difference in terms of age, body mass index, parathyroid hormone before parathyroidectomy, calcium before and after parathyroidectomy, and glomerular filtration rate. The multivariate linear regression model showed a negative association between body mass index, glomerular filtration rate, and total calcium after parathyroidectomy with parathyroid hormone variation. Patients with reduced glomerular filtration rate had higher levels of parathyroid hormone before parathyroidectomy, with no significant difference after the procedure between glomerular filtration rate groups.

**CONCLUSION:**

Persistently elevated levels of parathyroid hormone occurred in more than half of the patients subjected to parathyroidectomy. The observed variation in parathyroid hormone was significantly higher in patients with reduced glomerular filtration rate.

## INTRODUCTION

Primary hyperparathyroidism (PHPT) is a disorder of bone metabolism that excessively secretes the parathyroid hormone (PTH) from one or more parathyroid glands. Its prevalence is estimated at one to seven cases per 1,000 individuals and is considered the most common cause of ambulatorial hypercalcemia^1^.

PHPT has a variable clinical spectrum of presentation. The increase in bone resorption can lead to bone loss, fractures, osteitis fibrosa cystica, and the pathognomonic bone involvement of PHPT, characterized by "salt-and-pepper" lesions, periosteal resorption, bone cysts, and brown tumors^2^. Nephrolithiasis and nephrocalcinosis can also occur due to an increased renal flux of calcium. In addition to the classic bone and renal manifestations, PHPT may also be associated with cardiovascular disease and neurocognitive symptoms^3^. On the other hand, in countries where biochemical screening is routinely implemented, the most common presentation refers to asymptomatic PHPT with serum calcium no more than 1 mg/dL above the upper limit of the normal range^2^. The impact of asymptomatic PHPT on survival is uncertain, and surgical approach is recommended in situations of hypercalcemia and bone and kidney involvement^3^.

A successful surgery, defined as a normal calcium level 6 months after the procedure, occurs in more than 95% of cases^4^. In addition, it is expected that PTH levels drop dramatically, with post-surgery levels remaining within a normal range after parathyroidectomy (PTX). However, because of not well-understood factors, PTH levels can remain high even months after the procedure. Despite the established efficacy of the surgery, many patients show persistently elevated PTH (PePTH) levels after PTX, with a frequency varying from 3 to 46% and an average of 24%^5^. Reported risk factors for developing PePTH include older age, high preoperative PTH, larger adenomas, vitamin D deficiency, higher serum creatinine, and high bone turnover^4,5^.

Hyperparathyroidism and chronic kidney disease (CKD) are related conditions, but it is unclear whether reduced renal function in those with PHPT stems from the disease itself or from independent factors^3^. In fact, CKD progression is associated with higher levels of fibroblast growth factor 23 (FGF23), 1,25(OH)_2_ vitamin D deficiency, hypocalcemia, and hyperphosphatemia, factors that directly or indirectly increase PTH levels^6^. Higher PTH levels are associated with increased morbidity and mortality in CKD^7^.

The primary goal of this study was to determine the prevalence of PePTH after PTX in a tertiary hospital and to compare it with the available published data. Moreover, this research analyzed groups by age and CKD to infer the possible factors related to PePTH. This knowledge is relevant in clinical practice considering the prevalence of primary and secondary forms of hyperparathyroidism, the expansion of surgery indications, and the frequency of PePTH after parathyroid removal.

## METHODS

### Study population

A medical record query was performed using the medical record database of the institution to filter patients over 18 years of age who underwent PTX at Hospital de Clínicas de Porto Alegre, a tertiary hospital center in Southern Brazil, from January 2010 to December 2019. The query system was anonymized, and clinical, laboratory, and sociodemographic data were collected from all patients. The institutional ethics committee approved this study (CAAE 76718323.2.0000.5327), and consent forms were waived due to the anonymous nature of the accessed information. Patients subjected to PTX were identified by the standardized code SIGTAP 04.02.01.002-7, and all patients with at least one measurement of post-surgery PTH level were included. A review of all diagnoses associated with every chart (International Classification of Diseases, Tenth Revision [ICD-10]) was performed to exclude patients with genetic predisposition to hyperparathyroidism, such as multiple endocrine neoplasia. Only one procedure per patient was included in the analysis; if an individual was subjected to more than one surgery over the data collection period, only the last one was included in the analysis. Patients submitted to multiple interventions were not excluded. Individual characteristics included age, sex, weight, height, ethnic group, and date of surgery.

### Laboratory measurements

Laboratory results included serum levels of PTH, total calcium, 25(OH)D, creatinine, albumin, phosphorus, and alkaline phosphatase, both before and after surgery. The analyzed period included the 3 months prior to surgery and up to 18 months after surgery. If both periods showed multiple measurements, the median, maximum, minimum, and last values were captured to represent each period. The last post-surgery PTH level was considered to compare groups. The PTH variation levels were considered between pre- and post-surgery median values.

Circulating PTH and 25(OH)D levels were measured by chemiluminescent microparticle immunoassay (CMIA) (Abbott Alinity i). Total calcium, creatinine, albumin, and alkaline phosphatase were estimated colorimetrically (Abbott Alinity c). Corrected calcium was calculated by adding 0.8 mg/dL to the measured serum calcium for each g/dL under 4 mg/dL of measured serum albumin. Phosphorus was measured by ultraviolet (UV) phosphomolybdate (Abbott Alinity c).

Institutional laboratory measures considered normal were from 15 to 68 pg/mL, 30 to 60 ng/mL, 8.4 to 10.2 mg/dL, and 0.5 to 1.3 mg/dL for PTH, 25(OH)D, serum calcium, and serum creatinine, respectively. Inter- and intra-assay variability ranges were assumed as constant over the evaluated period. The multiple laboratory results of this study randomized the possible analytic error of each measurement.

### Statistical analysis

The Shapiro-Wilk normality test and descriptive statistics were used to assess data distribution. Results were shown as mean (standard deviation) or median (interquartile range), as applicable. Non-Gaussian variables were log-transformed for statistical analysis and then back-transformed to their original units of measure for reporting. Comparisons between group means were performed by a t-test or one-way analysis of variance.

Our primary outcome was the last PTH level up to 18 months after surgery. Patients with PePTH (above 68 pg/mL) were compared to patients with normal PTH levels (PnPTH). Univariate and multivariate linear regression analysis models were used to determine the independent effects of study variables on PTH levels considering the variation in PTH levels before and after surgery.

All analyses were made on IBM SPSS Statistics for Windows, version 18.0, and statistical differences were considered significant at p<0.05.

## RESULTS

Over the period of data collection, the chosen hospital performed 303 procedures in 293 patients, of which this study considered 261 in its final analysis—32 patients were excluded because of genetic predisposition to PHPT. Most patients were women (61.5%) and Caucasian (88.4%). The median age and body mass index (BMI) totaled 56 (45–65) years and 25.53 (22.19–28.86) kg/m^2^, respectively. In the post-surgery period, 117 patients showed PnPTH, whereas 144 patients, PePTH. Table 1 shows the comparison of the laboratory results for both groups.

**Table 1:** Clinical and laboratory features of 261 patients according to last measured post-surgery parathyroid hormone levels.

Variable	Elevated PTH (n=144)	Normal PTH (n=117)	p-value
Age (years)	57 (46–64.2)	54 (45.5–66)	0.452
BMI (kg/m^2^)	26.3 (22.0–28.9)	25.2 (22.7–28.9)	0.268
Median pre-surgery PTH (pg/mL)	491 (216–1,302)	419 (219–1,050)	0.352
Median post-surgery PTH (pg/mL)	104 (60–165)	26 (13–50)	**0.000**
Minimum post-surgery PTH (pg/mL)	23 (6–64)	4 (4–15)	**0.000**
Median pre-surgery calcium (mg/dL)	10.5±1.1	10.9±1.5	0.293
Median post-surgery calcium (mg/dL)	9.1±0.6	9.0±0.7	0.246
Pre-surgery GFR (mL/min/1.73m^2^)	47.2 (12.2–77)	48 (34–74.5)	0.336
Post-surgery GFR (mL/min/1.73m^2^)	40.5 (14.2–73.5)	38 (27–69)	0.231
Pre-surgery alkaline phosphatase (U/L)	147 (101–340)	139 (110–385)	0.688
Post-surgery alkaline phosphatase (U/L)	99 (73–190)	116 (90–200)	0.697
Pre-surgery phosphorus (mg/dL)	2.9 (2.3–4.1)	3.1 (2.4–3.8)	0.765
Post-surgery phosphorus (mg/dL)	3.2 (2.8–3.8)	3.5 (3.0–3.9)	0.120
Pre-surgery vitamin D (ng/mL)	23±11.8	19.3±6.2	0.147
Post-surgery vitamin D (ng/mL)	28.4±9.6	30±11.7	**0.010**

Values are shown as mean±SD or median (interquartile range). p-value according to the t-test. Statistically significant values are denoted in bold. BMI: body mass index; PTH: parathyroid hormone; GFR: glomerular filtration rate.

The PnPTH group showed lower median and minimum PTH levels after surgery and higher post-surgery 25(OH) D levels. Both groups showed no differences regarding age, BMI, pre-surgery PTH, corrected serum calcium, or pre- or post-surgery estimated GFR.

Univariate linear regression models demonstrated that pre-surgery PTH, GFR, and post-surgery calcium showed the greatest relation to the variation in PTH levels. Multivariate linear regression models showed that BMI, GFR, and post-surgery calcium levels were negatively associated with PTH variation in the studied period (p=0.000; R^2^=0.524). Adding age failed to improve the model (Table 2), although PePTH levels varied according to age. Patients younger than 50 years of age showed higher PePTH (51.3 [11.7–170.3]; 19.2 [6.6–47.9]; p=0.043) and higher values of creatinine (3.27 [1.43–7.81]; 1.45 [0.86–2.79]; p=0.000) than those older than 60 years.

**Table 2:** Multivariate linear regression model for pre- and post-surgery PTH variation levels.

Model (p<0.001 | R^2^=0.527)		
Variable	B (95%CI)	p
BMI (kg/m^2^)	-23.389 (-42.974, -3.805)	0.020
Post-surgery GFR (mL/min/1.73 m^2^)	-9.729 (-13.838, -5.620)	<0.001
Post-surgery calcium (mg/dL)	-340.753 (-477.881, -203.625)	<0.001
Age (years)	-2.622 (-11.272, 6.028)	0.548

PTH: parathyroid hormone; GFR: glomerular filtration rate; CI: confidence interval; BMI: body mass index.

**Figure 1:**
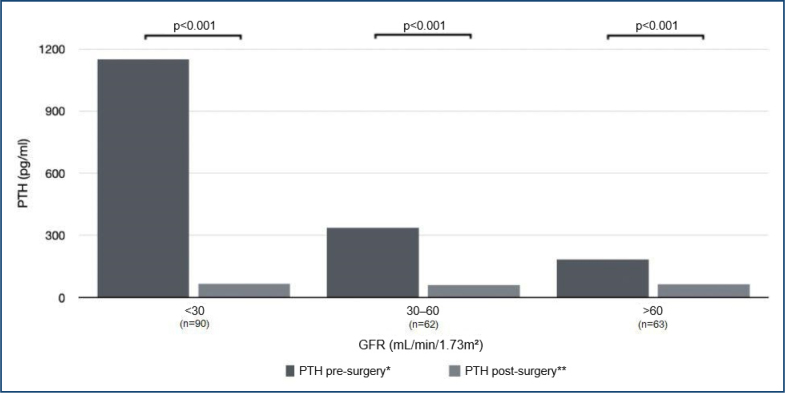
PTH variation after surgery according to glomerular filtration rate. p-value by t-test and one-way ANOVA. *Comparison among pre-surgery PTH levels according to glomerular filtration rate groups (p<0.001). **Comparison among post-surgery PTH levels according to glomerular filtration rate groups (p=0.119). PTH: parathyroid hormone; GFR: glomerular filtration rate.

The analysis according to GFR showed that patients with reduced GFR had greater variation in PTH levels after surgery (p<0.001) (Figure 1).

## DISCUSSION

In our study, 55% of the patients had PePTH levels after PTX according to the institutional reference values. Those who had normal PTH levels after PTX showed lower minimum PTH levels and higher values of vitamin D. Moreover, PTH variation after surgery was negatively associated with BMI, renal impairment, and post-surgery calcium. These findings suggest a complex interaction between parathyroid glands and kidney function, with patients in advanced CKD showing greater variation in PTH levels after surgery.

The prevalence of PePTH in our study was higher than previous findings in the literature, which ranged from 9 to 62%, with an average around 30%^4,5,8–15^. A possible explanation for the higher prevalence of PePTH in this study could stem from our population including patients with CKD, with a median of GFR lower than 60 mL/min/1.73m^2^. Most available studies excluded patients with CKD either by removing those in hemodialysis or subjected to kidney transplant^8,15^, having creatinine above 2 mg/dL^14^, or showing secondary or tertiary hyperparathyroidism^9–12^. Patients with CKD have higher levels of FGF23 and phosphorus and lower levels of vitamin D and calcium, resulting in higher levels of PTH by direct or indirect mechanisms^11^. In fact, previous data show that the risk of secondary hyperparathyroidism becomes apparent at GFRs<45 mL/min/1.73m^211^ and that levels of PTH in PHPT are more subtle in the 300–400-pg/mL range than in the 700–1,000-pg/mL one in patients with secondary hyperparathyroidism^16^.

To understand the relation between creatinine and PTH levels after surgery, this research stratified its results by the degree of renal function loss, with similar PTH levels after surgery, regardless of GFR. These findings suggest a possible primary parathyroid mechanism playing an important role in PTH levels before surgery, with a significant decrease even in patients with CKD. A possible explanation could refer to an improvement in renal function after PTX, but this study found no improvement in CKD after surgery. Another possibility could involve the progression for adynamic bone disease, a condition that may occur after PTX and that is characterized by decreased bone formation and low levels of PTH^17^.

Several factors are thought to be related with elevated PTH levels after surgery. The most established are higher levels of pre-surgery PTH^4,8,10–15^ and lower vitamin D^4,8,10,12,15^. Other variables described in previous studies include older age^8,14^, larger glands^8,10^, higher pre-surgery calcium^8^, higher alkaline phosphatase^8^, lower phosphorus^8,15^, higher creatinine^4,8,15^, higher BMI^10^, black skin color^10^, and male sex^11^. This study also related vitamin D to PePTH, finding no statistically significant differences regarding age, BMI, pre-surgery PTH, calcium, GFR, alkaline phosphatase, and phosphorus. However, when stratified by age categories, younger patients showed higher levels of PTH after PTX, a finding possibly stemming from the impaired kidney function in this group. As this study was carried out in a tertiary reference center, many patients with severe disease were referred to this hospital despite their younger age.

Carsello et al^10^ showed that higher BMI was positively associated with PTH levels, but those patients also had lower levels of vitamin D. Obesity and vitamin D are two well-correlated conditions. Considering a larger volume of distribution and the known link between vitamin D, fatty tissue, reduced absorption, and poor diets, individuals with obesity are at an established risk of vitamin D deficiency^18^. Moreover, Kota^8^ and Solorzano^14^ indicated that older age was related to PePTH, perhaps because of the expected decrease in renal function^19^ and vitamin D deficiency related to reduced sun exposure and limited production of the skin that characterize older adults^20^. In line with these results, Caldwell et al^4^ found that patients with PePTH had higher levels of creatinine, but this difference failed to remain significant after adjusting for age. In our study, these factors showed no statistical significance by themselves, but, when placed in a multivariate linear regression model, BMI, GFR, and total calcium after PTX showed a negative association with PTH variation in the analyzed period.

The limitations of this study include its restricted access to individual medical records based on the anonymized model, its lack of information regarding vitamin D or calcium supplementation, its inclusion of patients with CKD, and the loss of a few post-surgery laboratory measurements. Moreover, results on urinary calcium levels were available for very few patients, rendering reports unfeasible.

On the other hand, this study analyzed data from a reference center in Southern Brazil with a relevant sample size considering the available literature and analyzed a real-life population, in which CKD is very prevalent.

In conclusion, PePTH remains an important issue in osteometabolic health after PTX, especially in patients with CKD. More studies are necessary to establish the main causes and the clinical significance of elevated PTH levels after parathyroid removal, especially related to the fracture risk and cardiovascular disease in this context.

## Data Availability

The datasets generated and/or analyzed during the current study are available from the corresponding author upon reasonable request.
